# Supplementary material for: Taxonomic revision of the tarantula genus Aphonopelma Pocock, 1901 (Araneae, Mygalomorphae, Theraphosidae) within the United States
Source: Zookeys. 2016 Feb 4;(560):1–340. doi: 10.3897/zookeys.560.6264 (PMC4768370; doi:10.3897/zookeys.560.6264)
Supplement: Supplementary material 2 — Compressed files (.zip) containing Aphonopelma boxplot and PCA morphospace data. [file zookeys-560-001-s002.zip › Supp2a.zip!/Appendix 2A/PCA_morphospace/Aphonopelma_PCA_males/Sky_Islands/males_sky_PC1~PC2.pdf]

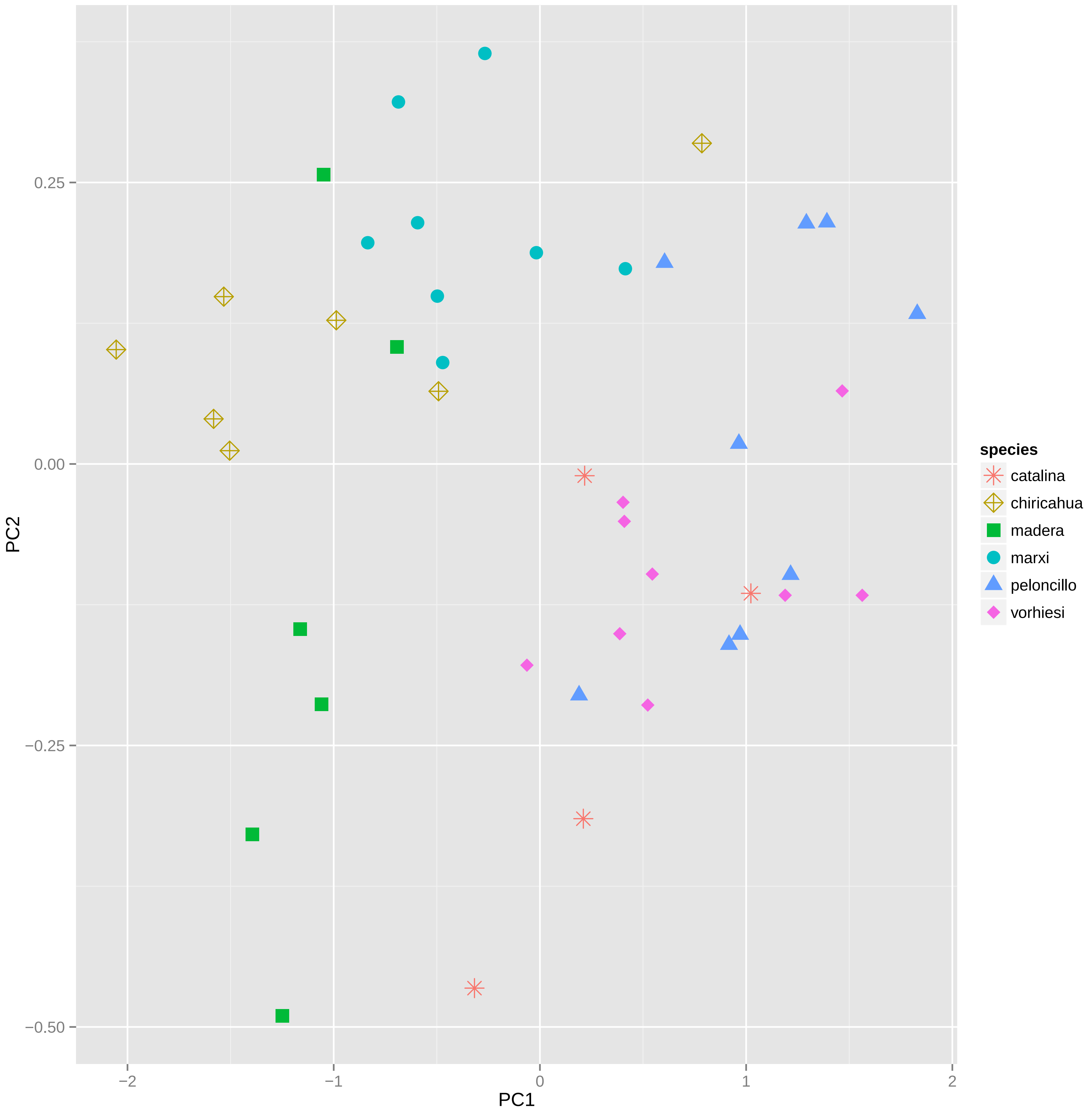

0.25
0.00
−0.25
−0.50
PC2
−2
−1
0
1
2
PC1
species
catalina
chiricahua
madera
marxi
peloncillo
vorhiesi
